# Incubation Period of COVID-19 Caused by Unique SARS-CoV-2 Strains: A Systematic Review and Meta-analysis

**DOI:** 10.1001/jamanetworkopen.2022.28008

**Published:** 2022-08-22

**Authors:** Yu Wu, Liangyu Kang, Zirui Guo, Jue Liu, Min Liu, Wannian Liang

**Affiliations:** 1 Department of Epidemiology and Biostatics, School of Public Health, Peking University, Beijing, China; 2 Vanke School of Public Health, Tsinghua University, Beijing, China

## Abstract

**Importance:**

Several studies were conducted to estimate the average incubation period of COVID-19; however, the incubation period of COVID-19 caused by different SARS-CoV-2 variants is not well described.

**Objective:**

To systematically assess the incubation period of COVID-19 and the incubation periods of COVID-19 caused by different SARS-CoV-2 variants in published studies.

**Data Sources:**

PubMed, EMBASE, and ScienceDirect were searched between December 1, 2019, and February 10, 2022.

**Study Selection:**

Original studies of the incubation period of COVID-19, defined as the time from infection to the onset of signs and symptoms.

**Data Extraction and Synthesis:**

Following the Preferred Reporting Items for Systematic Reviews and Meta-analyses (PRISMA) reporting guideline, 3 reviewers independently extracted the data from the eligible studies in March 2022. The parameters, or sufficient information to facilitate calculation of those values, were derived from random-effects meta-analysis.

**Main Outcomes and Measures:**

The mean estimate of the incubation period and different SARS-CoV-2 strains.

**Results:**

A total of 142 studies with 8112 patients were included. The pooled incubation period was 6.57 days (95% CI, 6.26-6.88) and ranged from 1.80 to 18.87 days. The incubation period of COVID-19 caused by the Alpha, Beta, Delta, and Omicron variants were reported in 1 study (with 6374 patients), 1 study (10 patients), 6 studies (2368 patients) and 5 studies (829 patients), respectively. The mean incubation period of COVID-19 was 5.00 days (95% CI, 4.94-5.06 days) for cases caused by the Alpha variant, 4.50 days (95% CI, 1.83-7.17 days) for the Beta variant, 4.41 days (95% CI, 3.76-5.05 days) for the Delta variant, and 3.42 days (95% CI, 2.88-3.96 days) for the Omicron variant. The mean incubation was 7.43 days (95% CI, 5.75-9.11 days) among older patients (ie, aged over 60 years old), 8.82 days (95% CI, 8.19-9.45 days) among infected children (ages 18 years or younger), 6.99 days (95% CI, 6.07-7.92 days) among patients with nonsevere illness, and 6.69 days (95% CI, 4.53-8.85 days) among patients with severe illness.

**Conclusions and Relevance:**

The findings of this study suggest that SARS-CoV-2 has evolved and mutated continuously throughout the COVID-19 pandemic, producing variants with different enhanced transmission and virulence. Identifying the incubation period of different variants is a key factor in determining the isolation period.

## Key Points

**Question:**

What are the incubation periods of COVID-19 caused by different SARS-CoV-2 strains?

**Findings:**

In this systematic review and meta-analysis of 141 articles, the pooled incubation period was 6.57 days. The incubation periods of COVID-19 caused by the Alpha, Beta, Delta, and Omicron variants were 5.00, 4.50, 4.41, and 3.42 days, respectively.

**Meaning:**

These results suggest that with the evolution of mutant strains, the incubation period of COVID-19 decreased gradually from Alpha variant to Omicron variant.

## Teaser

This systematic review and meta-analysis estimates the incubation periods of COVID-19 caused by various strains of SARS-CoV-2.

## Introduction

In December 2019, multiple cases of novel coronavirus disease (COVID-19), which is caused by severe acute respiratory syndrome coronavirus 2 (SARS-CoV-2), were reported in Wuhan, China.^1^ On March 11, 2020, the World Health Organization (WHO) declared that COVID-19 can be characterized as a pandemic. To date, the transmission of COVID-19 is still difficult to contain, as confirmed and death cases are still increasing. Up to March 16, 2022, 460 280 168 confirmed cases and 6 050 018 confirmed deaths have been reported to the WHO.^2^ Rapid spread of COVID-19 has had enormous social, economic, and health care system effects around the world. Effective treatment to block the spread of COVID-19 is not developed yet, so countries have implemented a series of nontreatment interventions such as social distancing, isolation, face mask mandates, and quarantining to reduce its rapid transmission.^3^ Existing evidence has shown that most of COVID-19 cases are missed by screening because infected persons are unaware they were exposed and have not developed symptoms yet.^4,5,6^

Incubation period is one of the most important epidemiological parameters of infectious diseases. Knowledge of the disease’s incubation period is of great significance for case definition, management of emerging threats, estimation of the duration of follow-up for contact tracing and secondary case detection, and the establishment of public health programs aimed at reducing local transmission.^7^ Previous studies^8^ have reported that the average serial interval of COVID-19 is shorter than the average incubation period, which suggests a substantial proportion of presymptomatic transmission. For diseases caused by different pathogens, the length of incubation period is the key factor to determine the isolation period of infected persons.

Since the beginning of the COVID-19 epidemic, SARS-CoV-2 has evolved and mutated continuously, producing variants with different transmissibility and virulence. SARS-CoV-2 variants are classified by the WHO into 2 types: variants of concern (VOC) and variants of interest (VOI).^9^ According to the US Center for Disease Control and Prevention (CDC), a VOC is a variant that has increased transmissibility, increased virulence, a resistance to vaccine or acquired immunity from previous infection, and has the ability to elude diagnostic detection.^10^ Several VOC have emerged from the original wild-type strain isolated in Wuhan since the outbreak first began in December 2019, such as Alpha (B.1.1.7), Beta (B.1.351), Gamma (P.1), Delta (B.1.617.2), and Omicron (B.1.1.529).^9^ The Alpha variant was first detected in the UK in September 2020; the Beta variant in South Africa in May 2020; and the Gamma variant in Brazil in September 2020. All 3 quickly became the main virus strains worldwide.

Globally, many studies were conducted to estimate the average incubation period of COVID-19. However, the reported estimates of incubation period in these fragmented studies vary depending on the number of study participants recruited, the type of design employed, the data collection period, and the country in which the study was conducted. In addition, with the spread of the Delta and Omicron variants, the current incubation period of COVID-19 is different from that in the outbreak of Wuhan. This meta-analysis was aimed to determine the overall pooled incubation period of COVID-19 and the incubation period of COVID-19 caused by different SARS-CoV-2 variants using available evidence, so as to adjust prevention and control strategies and better block the transmission of COVID-19.

## Methods

### Search Strategy

We conducted this meta-analysis following the Preferred Reporting Items for Systematic Reviews and Meta-Analyses (PRISMA) guideline. This review was not registered. This study was exempted from ethics review board at Peking University because it used previously published literature in its analysis. A survey of the literature was implemented between December 1, 2019, and February 10, 2022. Publications on the electronic databases PubMed, Embase and ScienceDirect were searched using the keywords *novel coronavirus*, *SARS-CoV-2*, *2019-nCoV*, or *COVID-19* and either *incubation period* or *incubation* (eTable 1 in the Supplement). No restrictions on language or publication status were imposed so long as an English abstract was available. The initial searches were carried out by 3 of the investigators (Y.W., L.K., R.G.).

### Inclusion and Exclusion Criteria

Inclusion criteria for selecting the studies were that the incubation period was one of the primary outcomes of the study and that, when the incubation periods of multiple groups were reported in the same study, only the group with the largest study population was included. Criteria for exclusion included articles not conducted as studies (ie, editorials, perspective articles, letters to the editor, reviews, article information, or comments), duplicate studies, and articles with overlapping study populations (ie, enrolling the same population in the same region around the same period).

### Outcome Measures and Study Selection

The outcome variable was the mean estimate of the incubation period. Incubation period was defined as the time from when the infection occurred to the onset of signs and symptoms or the first positive test. It was measured with cases of a well-defined period of exposure and symptom onset.

Results of searches were screened in 2 stages. First, titles and abstracts were screened and only relevant articles retained. Next, articles were read in detail—studies were selected for meta-analysis if they reported either results fitting our primary parameters (with CIs) or sufficient information to facilitate calculation of those values.

After screening for inclusion and exclusion criteria, data extraction was carried out from the included studies. The name of the first author, area of study, time period for data collection, characteristics of the study population, strain type, and estimates for the incubation period with 95% CI were extracted from the selected studies. Ninety-five percent CIs were estimated for the studies reporting mean with standard deviation by using the following formula, which is generally used to calculate the 95% CI for any parameter:



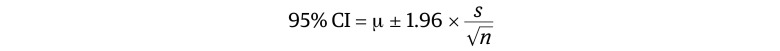



where *μ* indicates the mean incubation period, *s* the standard deviation, and *n* the sample size of the study. Some studies reported only median with interquartile range or range. Mean and the standard deviation were calculated for such studies by using an appropriate approximation for the consistency in synthesizing the results for meta-analysis.^11,12^

### Quality Assessment

Once studies were shortlisted, 2 authors (Y.W., L.K.) independently conducted appraisals of study quality. We used a scale modified from the Newcastle-Ottawa scale^13^ by McAloon et al^14^ to assess the quality of observational studies in meta-analyses (eTable 2 in the Supplement). This scale consists of 2 parts with a full score of 5 stars. The first part is external validity, with a maximum of 1 star; the second part is internal validity, which includes exposure window (a maximum of 2 stars) and outcomes with (a maximum of 2 stars). Based on the combined score of these 2 parts, each paper was categorized as either weak (1 star or less), moderate (2 to 3 stars), or strong (4 stars or more). After the studies were evaluated by the 2 authors, the results were compared and differences in ratings were resolved by discussion until a consensus rating was agreed upon.

### Statistical Analysis

A meta-analysis of continuous outcomes was employed for this study. We analyzed the data sets for the incubation period. After extracting all essential data using Excel 2021 (Microsoft Corporation), data were exported to Stata version 14.1 (StataCorp) statistical software for meta-analysis. A random-effect meta-analysis with an estimation of DerSimonian and Laird method was performed. Pooled mean estimates with 95% CIs were presented using forest plots. To determine the extent of variation between the studies, we conducted a heterogeneity test using the Higgins method, that was quantified by *I^2^* value.^15^ Publication bias was also assessed using a funnel plot. A 2-sided *P* < .05 was considered statistically significant.

## Results

### Search Results

**Figure 1.**
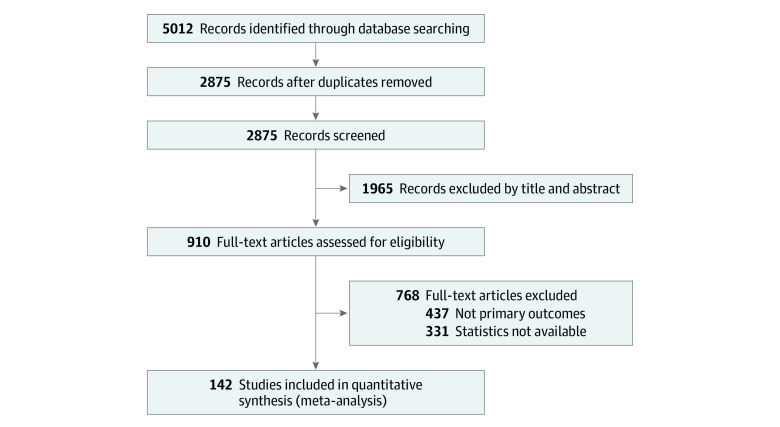
Study Flow Diagram

We identified 5012 records through PubMed, EMBASE, and Science Direct database searches, and documented the study selection process in a flowchart and showed the total numbers of retrieved references and the numbers of included and excluded studies (Figure 1). Based on the inclusion and exclusion criteria, 142 articles (8112 patients) were selected for analysis.^16,17,18,19,20,21,22,23,24,25,26,27,28,29,30,31,32,33,34,35,36,37,38,39,40,41,42,43,44,45,46,47,48,49,50,51,52,53,54,55,56,57,58,59,60,61,62,63,64,65,66,67,68,69,70,71,72,73,74,75,76,77,78,79,80,81,82,83,84,85,86,87,88,89,90,91,92,93,94,95,96,97,98,99,100,101,102,103,104,105,106,107,108,109,110,111,112,113,114,115,116,117,118,119,120,121,122,123,124,125,126,127,128,129,130,131,132,133,134,135,136,137,138,139,140,141,142,143,144,145,146,147,148,149,150,151,152,153,154,155,156,157^

### Study Characteristics

Over the 142 studies, the quality assessment gave 45 strong, 82 moderate, and 15 weak studies (eTable 3 in the Supplement). Most of the studies (93 [65.5%]) were conducted between January and March 2020 and most were conducted in China (108 [76.1%]). One study used case data from multiple countries around the world,^81^ 6 studies were conducted in South Korea,^37,46,51,52,59,124^ 4 in France,^25,29,35,113^ 3 in Japan,^80,105^ 2 in Singapore,^87,111^ 2 in India,^16,78,83,103^ 2 in Vietnam,^21,58^ and 2 in Australia.^45,99^ One hundred nineteen studies (83.8%) included patients infected with the wild-type strain, 5 (3.5%) with the multiple strains,^17,26,35,80,112^ and 11 (7.7%) with an unknown strain^84,93,99,106,116,126,135,142,148,150,157^ (eTable 4 in the Supplement).

### Pooled Average Estimate of Incubation Period

The mean incubation period of COVID-19 was 6.57 days (95% CI, 6.26-6.88 days), ranging from 1.80 to 18.87 days (Table). There was substantial heterogeneity between the studies (*I^2^* = 98.8%; *P* < .001). Our results suggested no potential publication bias in the included studies (eFigure 1 in the Supplement). The standard error for all the included studies in the meta-analysis was very low except for a 2020 study conducted by Xie^134^ where the highest standard error was observed.

**Table.** Mean Incubation Period of COVID-19 From Included Studies

Author	Mean incubation period (95% CI)	Weight
Areekal et al,^16^ 2021	4.22 (3.71-4.65)	0.81
Backer et al,^18^.2020	6.4 (5.6-7.7)	0.75
Backer et al,^17^ 2022	3.2 (2.93-3.47)	0.82
Bao et al,^19^ 2020	5.4 (4.5-6.3)	0.77
Brandal et al,^20^ 2021	3.33 (3.17-3.49)	0.82
Bui et al,^21^ 2020	6.4 (4.89-8.5)	0.64
Chen et al,^22^ 2020	8 (4.97-11.03)	0.46
Covid-Epidemiology Investigation Team,^23^ 2021	8.75 (6.95-10.55)	0.64
Dai et al,^24^ 2020	6.5 (5.9-7.1)	0.80
Del Águila-Mejía et al,^26^ 2022	3.1 (2.82-3.38)	0.82
Deng et al,^27^ 2021	8 (6.62-9.38)	0.71
Deng et al,^28^ 2021	9.1 (7.86-9.66)	0.77
Denis et al,^29^ 2021	4 (3.93-4.07)	0.82
Ding et al,^30^ 2020	7.11 (5.24-8.98)	0.63
Dong et al,^31^ 2020	7.25 (5.86-8.64)	0.70
Du et al,^32^ 2020	5.28 (4.75-5.82)	0.80
Gao et al,^34^ 2020	11.67 (9.46-13.87)	0.58
Gao et al,^33^ 2020	7.33 (3.54-11.13)	0.37
Grant et al,^35^ 2022	5 (4.95-5.05)	0.82
Guo et al,^36^ 2020	9.33 (8.21-10.46)	0.74
Han et al,^37^ 2020	6.63 (4.28-8.97)	0.56
Han et al,^39^ 2020	7.67 (7.08-8.25)	0.80
Han et al,^38^ 2020	5.5 (4.5-6.5)	0.76
Hong et al,^40^ 2020	5.7 (4.95-6.45)	0.78
Hua et al,^41^ 2020	9.1 (7.99-10.21)	0.74
Huang et al,^43^ 2020	8 (7.57-8.43)	0.81
Huang et al,^42^ 2020	5.5 (5.08-5.92)	0.81
Huang et al,^44^ 2021	7.8 (7.4-8.5)	0.80
Je et al,^45^ 2021	4.7 (3.21-6.19)	0.69
Jeong et al,^46^ 2020	5 (4.38-5.62)	0.79
Jiang et al,^47^ 2020	6.73 (5.97-7.48)	0.78
Jiang et al,^48^ 2021	7.75 (7.1-7.99)	0.81
Jin et al,^49^ 2020	5.33 (4.81-5.86)	0.80
Khonyongwa et al,^50^ 2020	6 (5.5-7)	0.78
Ki et al,^51^ 2020	5.39 (4.7-6.05)	0.79
Kim et al,^52^ 2020	11.86 (7.59-16.13)	0.32
Kong et al,^53^ 2020	6.33 (3.14-9.53)	0.44
Kong et al,^55^ 2020	8.5 (7.8-9.2)	0.79
Kong et al,^54^ 2020	7.25 (7.04-7.46)	0.82
Lai et al,^56^ 2020	7.67 (7.02-8.31)	0.79
Lau et al,^57^ 2021	4.75 (4.14-5.56)	0.79
Laval et al,^25^ 2021	4.61 (3.2-6.02)	0.70
Le et al,^58^ 2020	7 (4.87-9.13)	0.59
Lee et al,^59^ 2021	4.6 (4.33-4.87)	0.82
Lei et al,^60^ 2020	7.57 (3.95-11.19)	0.39
Leung et al,^61^ 2020	1.8 (1.63-1.97)	0.82
Li et al,^63^ 2020	5.33 (4.82-5.85)	0.80
Li et al,^64^ 2022	6.5 (5.86-7.2)	0.79
Li et al,^62^ 2020	5.2 (4.1-7)	0.70
Linton et al,^65^ 2020	5.6 (5-6.3)	0.79
Liu et al,^66^ 2021	13.5 (10.93-16.07)	0.52
Liu et al,^68^ 2020	6.67 (5.38-7.95)	0.72
Liu et al,^69^ 2020	6.02 (4.74-7.3)	0.72
Liu et al,^70^ 2020	6 (4.83-7.17)	0.73
Liu et al,^71^ 2020	8.8 (7.33-10.27)	0.69
Liu et al,^72^ 2020	9 (7.79-10.21)	0.73
Liu et al,^74^ 2021	8.4 (7.32-9.48)	0.75
Liu et al,^67^ 2020	6.35 (6.28-6.42)	0.82
Liu et al,^73^ 2020	7.67 (6.42-8.91)	0.72
Llaque-Quiroz et al,^75^ 2020	8.67 (5.76-11.58)	0.47
Mao et al,^76^ 2020	10.3 (8.18-12.42)	0.59
Moazzami et al,^77^ 2021	1.91 (1.24-2.59)	0.79
Ng et al,^78^ 2021	5.5 (4.99-6.01)	0.80
Nie et al,^79^ 2020	5 (4.84-5.16)	0.82
Ogata et al,^80^ 2022	3.7 (3.4-4)	0.81
Pak et al,^81^ 2020	6.6 (5.4-7.8)	0.73
Pan et al,^82^ 2020	6.11 (4.55-7.67)	0.68
Patrikar et al,^83^ 2020	6.93 (6.11-7.75)	0.78
Paul et al,^84^ 2021	6.74 (6.35-7.13)	0.81
Ping et al,^85^ 2021	6.48 (5.58-7.38)	0.77
Pongpirul et al,^86^ 2020	5.5 (4.69-6.31)	0.78
Pung et al,^87^ 2020	4.33 (3.25-5.41)	0.75
Qi et al,^88^ 2020	3.67 (2.87-4.46)	0.78
Qian et al,^89^ 2020	5.67 (4.89-6.44)	0.78
Qiu et al,^90^ 2020	11.25 (10.06-12.44)	0.73
Ratovoson et al,^91^ 2021	4.1 (0.7-7.5)	0.41
Ren et al,^92^ 2020	5.3 (4.6-6)	0.79
Samrah et al,^93^ 2021	6.33 (5.51-7.15)	0.78
Sanche et al,^94^ 2020	4.2 (3.5-5.1)	0.78
Shen et al,^95^ 2020	7.17 (3.34-11)	0.36
Shen et al,^96^ 2020	7.57 (5.41-9.73)	0.59
Shi et al,^97^ 2020	6.13 (2.95-9.32)	0.44
Shi et al,^98^ 2020	4.77 (3.61-5.94)	0.73
Shiel et al,^99^ 2021	5.33 (4.6-6.07)	0.78
Shu et al,^100^ 2020	5.17 (2.75-7.59)	0.55
Song et al,^101^ 2020	5.01 (4.31-5.69)	0.79
Song et al,^102^ 2020	8.23 (6.73-9.73)	0.69
Song et al,^103^ 2020	10 (8.54-11.46)	0.69
Su et al,^104^ 2021	5.4 (4.42-6.38)	0.76
Sugano et al,^105^ 2020	6.8 (5.57-8.03)	0.73
Sun et al,^106^ 2021	6.5 (4.55-8.45)	0.62
Sun et al,^107^ 2020	5.4 (4.88-5.92)	0.80
Sun et al,^108^ 2020	8.1 (6.73-9.47)	0.71
Sun et al,^109^ 2021	5.33 (1.93-8.73)	0.41
Sun et al,^110^ 2020	13 (9.53-16.47)	0.40
Tan et al,^111^ 2020	5.54 (5.18-5.9)	0.81
Tanaka et al,^112^ 2022	2.87 (2.56-3.17)	0.81
The SARS-CoV-2 variant with line,^113^ 2021	4.5 (1.83-7.17)	0.51
Tian et al,^114^ 2020	6.7 (6.07-7.33)	0.79
Tindale et al,^115^ 2020	8.68 (7.72-9.7)	0.76
Viego et al,^116^ 2020	7.9 (4.6-11.1)	0.43
Wang et al,^158^ 2021	10.64 (8.08-13.2)	0.52
Wang et al,^117^ 2020	6.5 (5.86-7.14)	0.79
Wang et al,^118^ 2020	6.3 (6-6.6)	0.81
Wang et al,^119^ 2020	4.5 (3-6.4)	0.66
Wang et al,^120^ 2020	6 (5.47-6.53)	0.80
Wei et al,^123^ 2021	8.8 (6.77-10.83)	0.61
Wei et al,^122^ 2020	5.67 (5.14-6.19)	0.80
Won et al,^124^ 2021	5.53 (3.98-8.09)	0.60
Wong et al,^125^ 2020	5.5 (4.05-6.95)	0.70
Wu et al,^128^ 2021	8.75 (7.51-9.99)	0.72
Wu et al,^127^ 2020	7 (4.9-9.1)	0.60
Wu et al,^126^ 2020	7 (5.78-8.22)	0.73
Wu et al,^129^ 2020	6.05 (4.87-7.23)	0.73
Xiao et al,^131^ 2020	7.18 (5.84-8.52)	0.71
Xiao et al,^133^ 2021	8.58 (7-9)	0.76
Xiao et al,^132^ 2020	8.98 (7.98-9.9)	0.76
Xiao et al,^130^ 2020	9.25 (8.78-9.72)	0.81
Xie et al,^134^ 2020	18.87 (9.01-28.73)	0.09
Xin et al,^135^ 2020	6.9 (6.3-7.5)	0.80
Xu et al,^136^ 2020	11.67 (9.87-13.47)	0.64
Xu et al,^137^ 2020	4 (3.6-4.4)	0.81
Yang et al,^138^ 2020	8.75 (8.39-9.11)	0.81
Yang et al,^139^ 2021	6.67 (5.64-7.7)	0.75
Yang et al,^140^ 2020	4 (1.33-6.67)	0.51
You et al,^141^ 2020	8 (7.28-8.72)	0.79
Yu et al,^143^ 2022	16.6 (16.22-16.98)	0.81
Yu et al,^142^ 2020	6.8 (6.23-7.37)	0.80
Zhang et al,^146^ 2021	4.3 (2.73-5.87)	0.68
Zhang et al,^144^ 2020	5.2 (1.8-12.4)	0.24
Zhang et al,^145^ 2020	6.75 (4.27-9.23)	0.54
Zhang et al,^147^ 2021	4.67 (3.92-5.41)	0.78
Zhang et al,^148^ 2021	6.1 (5.73-6.47)	0.81
Zhao et al,^151^ 2021	6.8 (6.2-7.5)	0.79
Zhao et al,^152^ 2020	7 (5.43-8.57)	0.68
Zhao et al,^153^ 2021	6.5 (5.6-7.4)	0.77
Zhao et al,^150^ 2021	4 (3.52-4.48)	0.80
Zhao et al,^149^ 2020	6.67 (4.86-8.48)	0.64
Zhong et al,^154^ 2020	6.85 (5.74-7.96)	0.74
Zhu et al,^156^ 2021	3.33 (2.81-3.85)	0.80
Zhu et al,^155^ 2020	7.27 (6.76-7.78)	0.80
Zhu et al,^157^ 2021	11.6 (10.6-12.7)	0.75
**Overall** ^a^	**6.57 (6.26-6.88)**	**100.00**

^a^ *I^2^* = 98.8%; *P* < .001.

### Mean Incubation Periods of COVID-19 Infected by Different Strains 

Across a total of 119 studies with data on the wild-type strain, the mean incubation period was 6.65 days (95% CI, 6.31-6.99) (eFigure 2 in the Supplement). For infections caused by the Alpha variant, an incubation period of 5.00 days (95% CI, 4.94-5.06) was reported in a single study.^35^ One study from France reported the incubation period of 4.50 days (95% CI, 1.83-7.17 days) for COVID-19 caused by the Beta variant.^113^ Another study reported the incubation period of COVID-19 caused by the Beta/Gamma variant was 5.10 days (95% CI, 4.87-5.33 days).^35^

**Figure 2.**
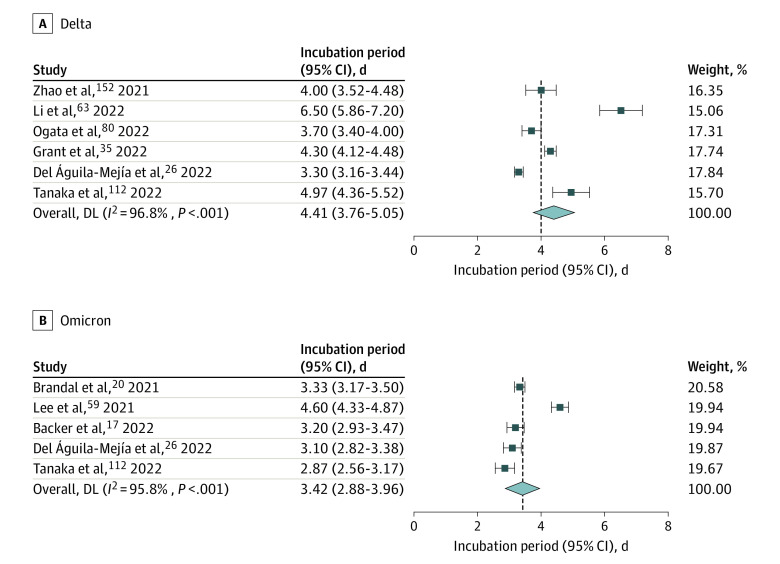
Forest Plot for Studies of Incubation Period of COVID-19 Caused by Different Variants

A total of 6 studies reported the incubation period of COVID-19 caused by the Delta variant, including 2 from China,^63,152^ 2 from Japan,^80,112^ 1 from France,^35^ and 1 from Spain,^26^ with a pooled incubation period of 4.41 days (95% CI, 3.76-5.05 days) (Figure 2). Five studies reported the incubation period of COVID-19 caused by the Omicron variant—1 each from Norway,^20^ Spain,^26^ Japan,^112^ the Netherlands,^17^ and South Korea^59^—with a pooled incubation period of 3.42 days (95% CI, 2.88-3.96 days) (Figure 2). With the evolution of the mutant strains, the incubation period of COVID-19 appeared to decrease gradually from the Alpha variant to Omicron variant, but there was no significant difference between the groups.

### Subgroup Analysis

**Figure 3.**
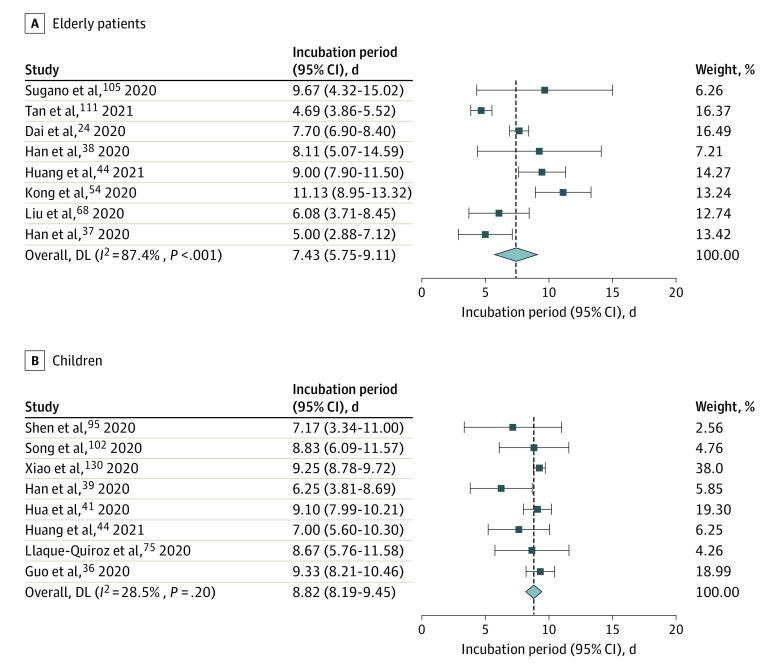
Incubation Period for COVID-19 in Older Patients and Infected Children Infectious strains were original strains.

A total of 8 studies reported the incubation period of COVID-19 among older patients (ie, aged 60 years or more).^24,37,38,44,54,68,105,111^ The pooled mean incubation period for these studies was 7.43 days (95% CI, 5.75-9.11 days), which was slightly higher than the pooled incubation period of the general population (6.65 days; 95% CI, 6.34-6.96 days), but the difference was not significant (Figure 3).

The mean incubation period of COVID-19 among infected children (under ages 18 years) was 8.82 days (95% CI, 8.19-9.45 days) across 8 studies,^36,39,41,44,75,95,102,130^ which was higher than the pooled incubation period of the general population (6.65 days; 95% CI, 6.34-6.96 days), and the difference was significant (*P* < .001) (Figure 3).

**Figure 4.**
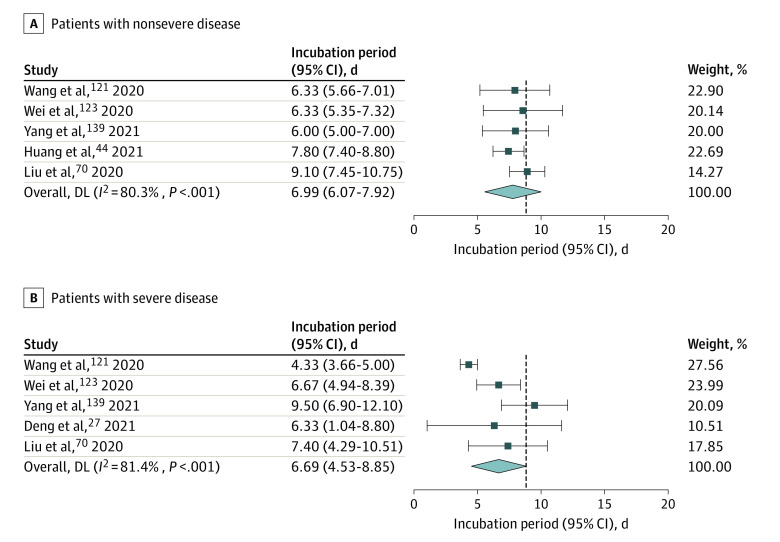
Incubation Period for COVID-19 in Patients With Severe and Nonsevere Illness Infectious strains were original strains.

Five studies reported the incubation period in patients with nonsevere illness,^44,70,121,123,139^ with a pooled value of 6.99 days (95% CI, 6.07-7.92 days). Five studies analyzed the incubation period of patients with severe disease,^27,70,121,123,139^ with a pooled value of 6.69 days (95% CI, 4.53-8.85 days), which was slightly shorter than that of patients with nonsevere illness, but the difference was not significant (Figure 4).

## Discussion

Our findings suggested that COVID-19 had a mean incubation period of 6.57 days (95% CI, 6.26-6.88 days), which was similar to the results of Elias et al^159^ (6.38 days; 95% CI, 5.79-6.97 days) and McAloon et al^14^ (6.5 days, 95% CI, 5.9-7.1 days). COVID-19 seemed to have a longer incubation period than that of other acute respiratory viral infections such as human coronavirus (3.2 days), influenza A (1.43-1.64 days), parainfluenza (2.6 days), respiratory syncytial virus (4.4 days), and rhinovirus (1.4 days).^160^ Furthermore, the median incubation period for SARS in 2009 had been estimated as 4.0 days,^160^ which was lower than COVID-19. In this study, the shortest mean incubation reported was 1.8 days and the longest incubation was 18.87 days. At present, based on the assumption that the incubation period of COVID-19 is 1 to 14 days, the WHO still recommends that COVID-19 close contacts be isolated for 14 days.^161^

Our study found that the incubation period of COVID-19 caused by Alpha variant was 5.00 days (95% CI, 4.94-5.06 days), and the incubation period of COVID-19 caused by Beta variant was 4.50 days (95% CI, 1.83-7.17 days), which were similar to that of the wild-type strain in Wuhan, China (5.2 days).^64,145^

The Delta variant, which was first reported in India in October 2020, was dominant in the second wave of COVID-19 outbreak in India in May 2021.^9^ Our study revealed that the incubation period of COVID-19 caused by Delta variant was 4.41 days (95% CI, 3.76-5.05), which was shorter than the pooled incubation period of COVID-19 (6.26 days), and also shorter than that caused by Alpha variant and Beta variants.

On November 24, 2021, South Africa first discovered and reported a case of Omicron variant infection to the WHO. Since then, this variant has quickly become the main virus strain in South Africa and spread to many countries and regions around the world. The Omicron variant is exceptional for carrying over 30 mutations in the spike glycoprotein, which have been predicted to influence antibody neutralization and spike function.^162^ Our study revealed that the incubation period of COVID-19 caused by Omicron variant was 3.42 days (95% CI, 2.88-3.96 days), which was shorter than the Alpha, Beta, and Delta variants. The CDC released new quarantine and isolation policy on March 30, 2022, which stated that people exposed to COVID-19 should stay home and away from other people for at least 5 days.^163^ At present, some countries around the world require close contacts to be isolated for 14 days. However, with the shortening of the incubation period of new variants, the isolation period can be adjusted appropriately to reduce the pressure on the health system.

Eight studies reported the incubation period among older patients (ages 60 years and older), and the mean incubation period of older patients was about 7.43 days (95% CI, 5.75-9.11), which was slightly higher than the pooled incubation period among the general population. Although the difference between the incubation periods of older patients and the overall incubation period was not significant, there was still a lot of evidence to support the hypothesis of a longer incubation period in older populations due to a slower immune response among older patients. Cowling et al^164^ hypothesized about this in their report on SARS in 2007, where they demonstrated that older patients had longer incubation periods, suggesting that this might have resulted from a delayed immune response. A study by Chen et al^165^ revealed that several SARS-CoV nonstructural proteins that were shared by SARS-CoV-2 suppress the type 1 interferon response, and such suppression was shown to lead to poor CD8^+^ T-cell response to viral infection. Therefore, age-associated weaker type 1 interferon responses coupled with direct viral suppression could serve as a critical innate immune mechanism that leads to poor cell-mediated immunity and increased vulnerability of older adults to SARS-CoV-2 infection with therapeutic implication. Additionally, older patients were more likely to experience symptom minimization and be more likely to ignore early symptoms and only report later when symptoms become more severe or intolerable.^111^ The lack of a fever response in older patients, the nonspecific geriatric presentations in an infectious illness (such as falls and delirium), and multi-comorbidities might result in a delayed awareness of disease onset and its detection by a clinician.^54^

Additionally, our study also revealed that the mean incubation period for infected children (8.82 days; 95% CI, 8.19-9.45) was also shorter than the pooled incubation period among the general population (6.65 days). Infected children tend to present with mild clinical symptoms without the classic phenotype of lung pneumonia, and COVID-19 symptoms are easily confused with other influenza-like illnesses, which renders infected children difficult to identify.^130^ Second, previous studies found that children can be a source of transmission during the viral incubation period. Some infected children may have an incubation period of more than 14 days. Indeed, it is difficult for investigators to collect information about the symptoms of very young children because they cannot accurately express their symptoms.^130^

Previous studies on SARS indicated that the incubation period of patients was related to the severity of the disease, and the incubation period of fatal cases was shorter.^166^ Virlogeux et al^167^ also found that Middle East Respiratory Syndrome patients with a shorter incubation period proceeded to have more severe disease. However, there are few studies on the association between the length of COVID-19 incubation period and the severity of infection. Our study found that the incubation period of COVID-19 in patients with severe illness was shorter (6.69 days) than patients with nonsevere illness. Studies have indicated that shorter incubation periods are associated with more serious disease, and this is related to the number of cells initially infected by the virus.^123^

This study was the first meta-analysis of the incubation period of COVID-19 caused by SARS-CoV-2 variants. We compared the incubation period of COVID-19 caused by different variants and the wild-type strain, and the results may be helpful in changing public health guidance on duration of quarantine, outbreak investigation, and contact tracing.

### Limitations

This study had several limitations. First, by definition, the required case data for the determination of individual incubation periods need to include both exposure (window) and onset of symptoms. In most studies, the data were collected retrospectively, resulting in a recall bias (uncertain exact dates of exposure) that would inevitably influence our assessment. Second, the estimate of the incubation period was computed with data with considerable heterogeneity. Possible sources of heterogeneity included difference in study population, data collection period, and method of analysis. Wild-type strain studies were mostly from Chinese patients; while variants studies were not. Population factors, especially those related to public policy and social behavior, may be confounding variables. In this study, we assumed that the incubation period was consistent across populations. Third, there were few studies on the incubation period of COVID-19 caused by SARS-CoV-2 variants. Because of the urgent timeline for data extraction and analysis, these studies have estimated the incubation period in a limited case number in a short period of time, which necessitates the cautious interpretation of the generalizability of our findings. The numbers were too small to detect systematic differences in incubation time in regards to age or sex.

## Conclusions

Although variants such as Alpha, Beta, and Gamma are currently only prevalent in a few countries in Southeast Asia, South America, and Africa, the Delta and Omicron variants have become the dominant strains in many countries around the world. Identifying the incubation period of different variants is a key factor in determining the isolation period. The pooled incubation period of COVID-19 in this study was 6.57 days. The incubation period for COVID-19 caused by the Alpha and Beta variants was approximately 5 days. The incubation period of COVID-19 caused by the Delta and Omicron variants was significantly shorter than that of the other variants.

## Notes

Supplement. **eTable 1.** Search Strategies for Meta-analysis of Observational Studies Reporting the Incubation Period of COVID-19 **eTable 2.** Quality Assessment Scale **eTable 3.** Quality Assessment of Final Studies Used in the Meta-analysis of Incubation Period **eTable 4.** Characteristics of the Studies Included in the Systematic Review and Meta-analysis **eFigure 1.** Funnel Plot with a 95% Confidence Interval for Included Studies in the Meta-analysis **eFigure 2.** Forest Plot for the Meta-analysis of Incubation Period for COVID-19 Caused by Wild-type Strain
