# Error in Discussion

**DOI:** 10.1001/jamanetworkopen.2022.35424

**Published:** 2022-09-16

In the Original Investigation titled “Incubation Period of COVID-19 Caused by Unique SARS-CoV-2 Strains: A Systematic Review and Meta-analysis,”^1^ published August 22, 2022, there was an error in the language describing the mean incubation period of COVID-19 among infected children vs the general population, which should have been described as longer. This article has been corrected.^1^
