# Supplementary material for: Incubation Period of COVID-19 Caused by Unique SARS-CoV-2 Strains: A Systematic Review and Meta-analysis
Source: JAMA Netw Open. 2022 Aug 22;5(8):e2228008. doi: 10.1001/jamanetworkopen.2022.28008 (PMC9396366; doi:10.1001/jamanetworkopen.2022.28008)
Supplement: Supplement. — eTable 1. Search Strategies for Meta-analysis of Observational Studies Reporting the Incubation Period of COVID-19 eTable 2. Quality Assessment Scale eTable 3. Quality Assessment of Final Studies Used in the Meta-analysis of Incubation Period eTable 4. Characteristics of the Studies Included in the Systematic Review and Meta-analysis eFigure 1. Funnel Plot with a 95% Confidence Interval for Included Studies in the Meta-analysis eFigure 2. Forest Plot for the Meta-analysis of Incubation Period for COVID-19 Caused by Wild-type Strain [file jamanetwopen-e2228008-s001.pdf]

# Supplemental Online Content

Wu Y, Kang L, Guo Z, Liu J, Liu M, Liang W. Incubation period of COVID-19 caused by unique SARS-CoV-2 strains. *JAMA Netw Open.* 2022;5(8):e2228008. doi:10.1001/jamanetworkopen.2022.28008

**eTable 1.** Search Strategies for Meta-analysis of Observational Studies Reporting the Incubation Period of COVID-19

**eTable 2.** Quality Assessment Scale

**eTable 3.** Quality Assessment of Final Studies Used in the Meta-analysis of Incubation Period

**eTable 4.** Characteristics of the Studies Included in the Systematic Review and Meta-analysis

**eFigure 1.** Funnel Plot with a 95% Confidence Interval for Included Studies in the Meta-analysis

**eFigure 2.** Forest Plot for the Meta-analysis of Incubation Period for COVID-19 Caused by Wild-type Strain

This supplemental material has been provided by the authors to give readers additional information about their work.

© 2022 Wu Y et al. *JAMA Network Open.*

**eTable 1. Search Strategies for Meta-analysis of Observational Studies Reporting the Incubation Period of COVID-19**

| Database | Search strategy |
|---|---|
| PubMed | ("Coronavirus"[MeSH Terms] OR "Coronavirus Infections"[MeSH Terms] OR "COVID-19"[Supplementary Concept] OR "coronavir*"[Title/Abstract] OR "corona virus*"[Title/Abstract] OR "covid"[Title/Abstract] OR "nCoV"[Title/Abstract] OR "2019 novel*"[Title/Abstract] OR "2019nCoV"[Title/Abstract] OR "2019-CoV"[Title/Abstract] OR "nCoV2019"[Title/Abstract] OR "pneumonia virus*"[Title/Abstract] OR "Severe Acute Respiratory Syndrome"[Title/Abstract] OR "SARS"[Title/Abstract] OR "sarscov*"[Title/Abstract]) AND ("incubation period"[Text Word] OR "incubation"[Text Word]) |
| EMBASE | ('covid 19'/exp OR 'coronavirus disease 2019'/exp OR 'severe acute respiratory syndrome coronavirus 2'/exp OR 'SARS-related coronavirus'/exp OR 'coronaviridae'/de OR 'coronavirinae'/exp OR 'coronavirus infection'/exp OR (coronavir* OR 'corona virus*' OR covid OR COVID19 OR nCoV OR '2019-novel*' OR 2019nCoV OR 2019 - CoV OR nCoV2019 OR 'pneumonia virus*' OR 'Severe Acute Respiratory Syndrome*' OR SARS OR SARSCoV*):ab,ti,kw) AND ('incubation period' OR incubation) |
| Web of science | TS = (((coronavir* OR "corona virus*" OR covid OR COVID19 OR nCoV OR "2019-novel Cov" OR 2019nCoV OR 2019 - CoV OR nCoV2019 OR "pneumonia virus*" OR "Severe Acute Respiratory Syndrome" OR SARS OR SARS-CoV-2)) AND (("incubation period" OR incubation))) |

**eTable 2. Quality Assessment Scale**

| Part | |
|---|---|
| **External validity** | 1) Representativeness of the study cohort<br>a) No selection of cases based on age, sex or general health status, supported by descriptive statistics demonstrating comparability with overall population※<br>b) No selection of cases based on age, sex or general health status, not supported by descriptive statistics※<br>c) Cases are likely to be biased towards those with more severe COVID-19 symptoms due to selection process – e.g. records from hospitalised patients<br>d) Cases are selected (e.g. based on age or sex) to represent a particular cohort of individuals<br>e) No description of the derivation of the cohort |
| **Internal validity** | **Exposure window**<br>2) Ascertainment of exposure<br>a) original data collected through interview ※<br>b) travel period only ※<br>c) secondary data (using publicly available reports)<br><br>3) Precision of the exposure window for cases used in final analysis<br>a) only includes cases with a 1-day exposure window ※<br>b) only includes cases with less than or equal to 3-day exposure window<br>c) includes cases with a range of exposure windows but statistical methods are used to account for this<br>d) includes cases with a range of exposure windows<br>e) no description/not clear |
| | **Outcome**<br>4) Assessment of outcome (onset of symptoms)<br>a) original data collected through interview ※<br>b) no description/not clear<br><br>5) Precision of estimate of outcome<br>a) Precise date ※<br>b) Window<br>c) no description/not clear |

© 2022 Wu Y et al. *JAMA Network Open.*

## eTable 3. Quality Assessment of Final Studies Used in the Meta-analysis of Incubation Period

| Author | Area | Time period | Quality assessment item category | | | | | Quality assessment |
|---|---|---|---|---|---|---|---|---|
| | | | 1 | 2 | 3 | 4 | 5 | |
| Areekal et al [16], 2021 | India | 2020.06-2020.07 | a | a | e | a | c | Moderate |
| Backer et al [18], 2020 | China | 2020.01.20-2020.01.28 | a | c | d | b | a | Moderate |
| Backer et al [17], 2020 | Netherlands | 2021.12.13-2021.12.26 | a | a | c | a | a | Strong |
| Bao et al [19], 2020 | China | 2020.01.15-2020.02.06 | a | a | a | a | a | Strong |
| | | | | | | | | |
| Brandal et al [20], 2021 | Norway | 2021.11.26-2021.12.13 | a | a | a | a | a | Strong |
| Bui et al [21], 2020 | Vietnam | 2020.01.23-2020.04.13 | a | c | c | b | a | Moderate |
| Chen et al [22], 2020 | China | 2020.01.24-2020.02.13 | c | a | d | a | c | Moderate |
| Covid-Epidemiology Investigation Team [23], 2021 | China | 2020.12.23-2021.01.31 | a | a | c | a | a | Strong |
| Dai et al [24], 2020 | China | 2020.01.20-2020.02.29 | a | b | d | a | a | Moderate |
| Del Águila-Mejía et al [26], 2022 | Spain | 2021.11-2021.12 | a | a | a | a | a | Strong |
| Deng et al [27], 2021 | China | by 2020.03.09 | c | a | a | a | a | Strong |
| Deng et al [28], 2020 | China | by 2020.03.31 | a | c | e | a | a | Moderate |
| Denis et al [29], 2021 | France | 2020.12.15-2020.12.24 | a | a | e | a | c | Moderate |
| Ding et al [30], 2020 | China | 2020.01.21-2020.03.10 | a | a | a | a | a | Strong |
| Dong et al [31], 2020 | China | 2020.01.13-2020.02.21 | a | c | a | b | a | Moderate |
| Du et al [32], 2020 | China | 2020.01.08-2020.02.05 | a | c | d | b | b | Weak |
| Gao et al [34], 2020 | China | 2020.01.21-2020.02.11 | a | c | a | b | a | Moderate |
| Gao et al [33], 2020 | China | 2020.01.20-2020.03.17 | c | a | e | a | b | Moderate |
| Grant et al [35], 2022 | France | 2021.05.23-2021.08.13 | a | a | a | a | a | Strong |
| Guo et al [36], 2020 | China | 2020.01.15-2020.03.15 | c | c | a | b | a | Moderate |
| Han et al [37], 2020 | South Korea | 2020.03.28-2020.04.08 | a | a | d | a | a | Strong |
| Han et al [39], 2020 | China | 2020.01.20-2020.03.10 | a | a | c | b | a | Moderate |
| Han et al [38], 2020 | China | 2020.01.31-2020.02.16 | c | a | e | a | a | Moderate |
| Hong et al [40], 2020 | China | 2020.01.14-2020.03.09 | a | c | a | b | a | Moderate |
| Hua et al [41], 2020 | China | 2020.01.01-2020.02.29 | d | a | b | b | a | Moderate |
| Huang et al [43], 2020 | China | 2020.01.08-2020.02.16 | a | c | c | b | c | Weak |
| Huang et al [42], 2020 | China | 2020.01.22-2020.06.18 | a | c | a | b | a | Moderate |
| Huang et al [44], 2021 | China | 2020.01.01-2020.04.08 | a | c | d | b | a | Moderate |
| Je et al [45], 2021 | Australia | 2020.02-2020.04 | a | c | d | b | a | Moderate |
| Jeong et al [46], 2020 | South Korea | 2020.02.22-2020.02.26 | c | a | a | a | a | Strong |
| Jiang et al [47], 2020 | China | 2020.01.12-2020.02.20 | a | a | e | a | a | Strong |
| Jiang et al [48], 2021 | China | 2020.01.19-2020.01.23 | a | c | c | b | a | Moderate |
| Jin et al [49], 2020 | China | 2020.01.17-2020.02.08 | a | b | a | b | a | Moderate |
| Khonyongwa et al [50], 2020 | UK | 2020.03.01-2020.04.18 | c | a | c | a | a | Moderate |
| Ki et al [51], 2020 | South Korea | 2020.03.01-2020.06.30 | a | c | d | b | b | Weak |
| Kim et al [52], 2020 | South Korea | 2020.02.04-2020.04.07 | c | a | a | a | a | Strong |

© 2022 Wu Y et al. *JAMA Network Open.*

| | | | | | | | | |
|---|---|---|---|---|---|---|---|---|
| Kong et al [53], 2020 | China | 2020.01.08-2020.01.27 | a | c | e | a | a | Moderate |
| Kong et al [55], 2020 | China | 2020.01.22-2020.02.15 | d | e | b | b | a | Weak |
| Kong et al [54], 2020 | China | 2020.01.25-2020.02.20 | a | a | c | b | a | Moderate |
| Lai et al [56], 2020 | China | 2020.01.11-2020.02.10 | c | c | e | a | a | Moderate |
| Lau et al [57], 2021 | China | 2020.01.01-2020.01.29 | a | c | c | b | b | Weak |
| Laval et al [25], 2021 | France | 2020.02.25-2020.03.04 | a | a | e | a | a | Strong |
| Le et al [58], 2020 | Vietnam | 2020.01-2020.02 | a | a | c | a | a | Strong |
| Lee et al [59], 2021 | South Korea | 2021.11.24-2021.12.10 | a | a | d | a | a | Strong |
| Lei et al [60], 2020 | China | 2020.01.22.2020.02.01 | d | a | d | a | a | Moderate |
| Leung et al [61], 2020 | China | 2020.01.20-2020.02.12 | a | c | d | b | a | Moderate |
| Li et al [63], 2020 | China | 2020.01.21-2020.02.09 | c | a | d | a | c | Moderate |
| Li et al [64], 2022 | China | 2021.05.21-2021.06.24 | a | c | a | b | a | Moderate |
| Li et al [62], 2020 | China | 2019.12-2020.01 | a | a | a | a | a | Strong |
| Linton et al [65], 2020 | China | by 2020.01.31 | a | c | d | b | a | Moderate |
| Liu et al [66], 2021 | China | 2020.01.20-2020.02.23 | a | a | d | a | a | Strong |
| Liu et al [68], 2020 | China | 2020.01.20-2020.04.15 | c | a | a | a | a | Strong |
| Liu et al [69], 2020 | China | 2019.12.25-2020.02.05 | a | a | a | a | a | Strong |
| Liu et al [70], 2020 | China | 2020.01.28-2020.04.12 | a | c | e | b | a | Moderate |
| Liu et al [71], 2020 | China | 2020.01.22-2020.02.21 | a | a | a | a | a | Moderate |
| Liu et al [72], 2020 | China | 2020.01.23-2020.02.10 | c | a | e | a | c | Strong |
| Liu et al [74], 2021 | China | 2020.01.01-2020.03.30 | a | a | a | a | a | Strong |
| Liu et al [67], 2020 | China | by 2020.02.11 | a | c | c | b | a | Moderate |
| Liu et al [73], 2020 | China | 2020.01.20-2020.03.17 | c | a | e | a | a | Moderate |
| Llaque-Quiroz et al [75], 2020 | Peru | 2020.03-2020.05 | d | a | e | a | a | Moderate |
| Mao et al [76], 2020 | China | by 2020.03.17 | a | c | a | b | a | Moderate |
| Moazzami et al [77], 2021 | Iran | 2020.04 | c | a | c | a | a | Moderate |
| Ng et al [78], 2021 | China | by 2020.03.21 | a | c | e | b | a | Moderate |
| Nie et al [79], 2020 | China | 2020.01.19-2020.02.08 | a | c | c | b | a | Moderate |
| Ogata et al [80], 2022 | Japan | 2020.07.01-2021.09.16 | a | a | a | a | a | Strong |
| Pak et al [81], 2020 | Global | 2019.12.01-2020.03.30 | a | c | c | b | b | Weak |
| Pan et al [82], 2020 | China | 2020.01-2020.02.20 | a | a | a | a | a | Strong |
| Patrikar et al [83], 2020 | India | by 2020.05 | a | c | a | b | c | Moderate |
| Paul et al [84], 2021 | Canada | by 2020.11.07 | b | c | e | b | c | Weak |
| Ping et al [85], 2021 | China | 2020.01.03-2020.03.05 | a | a | a | a | a | Strong |
| Pongpirul et al [86], 2020 | Thailand | 2020.01.08-2020.04.16 | a | a | a | a | a | Strong |
| Pung et al [87], 2020 | Singapore | 2020.02.03-2020.02.15 | a | a | e | a | c | Moderate |
| Qi et al [88], 2020 | China | 2020.01.13-2020.03.23 | c | a | c | a | b | Moderate |
| Qian et al [89], 2020 | China | 2020.01.20-2020.02.11 | a | a | e | a | c | Moderate |
| Qiu et al [90], 2020 | China | 2020.01.22-2020.02.12 | c | a | e | a | c | Moderate |
| Ratovoson et al [91], 2021 | Madagascar | 2020.03.09-2020.07.30 | a | a | c | a | a | Strong |
| Ren et al [92], 2020 | China | by 2020.01.23 | a | c | d | b | a | Moderate |
| Samrah et al [93], 2021 | Jordan | 2020.03.15-2020.06.09 | c | b | a | a | a | Strong |

© 2022 Wu Y et al. *JAMA Network Open.*

| | | | | | | | | |
|---|---|---|---|---|---|---|---|---|
| Sanche et al [94], 2020 | China | 2020.01.15-2020.01.30 | a | c | a | b | a | Moderate |
| Shen et al [95], 2020 | China | 2020.01.08-2020.02.19 | d | c | a | a | a | Moderate |
| Shen et al [96], 2020 | China | 2020.01-2020.02 | a | b | b | a | b | Moderate |
| Shi et al [97], 2020 | China | 2020.01.17-2020.03.05 | c | b | b | a | a | Moderate |
| Shi et al [98], 2020 | China | 2020.01.18-2020.03.02 | a | a | c | a | a | Strong |
| Shiel et al [99], 2021 | Australia | 2020.01-2020.06 | a | c | a | a | a | Strong |
| Shu et al [100], 2020 | China | by 2020.02.28 | c | a | d | a | c | Moderate |
| Song et al [101], 2020 | China | by 2020.01.26 | b | c | c | b | a | Moderate |
| Song et al [102], 2020 | China | 2020.01.16-2020.01.29 | a | a | d | a | a | Strong |
| Song et al [103], 2020 | China | 2020.02-2020.03 | c | a | e | b | c | Weak |
| Su et al [104], 2021 | China | 2020.01.21-2020.04.18 | a | c | e | b | c | Weak |
| Sugano et al [105], 2020 | Japan | 2020.01.15-2020.03.19 | a | c | a | b | a | Moderate |
| Sun et al [106], 2021 | China | 2020.12.23-2021.01.10 | a | a | e | b | c | Moderate |
| Sun et al [107], 2020 | China | 2020.01.24-2020.02.16 | a | a | e | a | c | Moderate |
| Sun et al [108], 2020 | China | 2020.01.20-2020.02.15 | c | a | e | a | a | Moderate |
| Sun et al [109], 2021 | China | 2020.06-2020.07.10 | a | b | a | b | a | Strong |
| Sun et al [110], 2020 | China | 2020.02 | c | c | a | a | b | Moderate |
| Tan et al [111], 2020 | Singapore | 2020.01.23-2020.04.02 | a | a | a | a | b | Strong |
| Tanaka et al [112], 2022 | Japan | 2022.01.01-2022.01.28 | a | a | c | a | a | Strong |
| The SARS-CoV-2 variant with line [113], 2021 | France | 2020.12-2021.01 | a | a | e | a | a | Strong |
| Tian et al [114], 2020 | China | 2020.01.20-2020.02.10 | c | a | e | a | c | Moderate |
| Tindale et al [115], 2020 | China | 2020.01.21-2020.02.22 | b | c | b | b | c | Weak |
| Viego et al [116], 2020 | Argentina | 2020.03.20-2020.05.08 | b | a | e | b | c | Moderate |
| Wang et al [158], 2021 | China | by2020.02.26 | a | a | a | a | a | Strong |
| Wang et al [117], 2020 | China | 2020.01.23-2020.02.20 | a | a | e | a | c | Moderate |
| Wang et al [118], 2020 | China | 2020.01.01-2020.04.03 | a | a | e | a | c | Moderate |
| Wang et al [119], 2020 | China | 2020.01.05-2020.02.12 | a | a | a | a | a | Strong |
| Wang et al [120], 2020 | China | 2020.01.20-2020.02.10 | c | a | e | a | c | Moderate |
| Wei et al [123], 2021 | China | 2020.01.19-2020.03.01 | c | a | e | b | c | Weak |
| Wei et al [122], 2020 | China | 2020.01.27-2020.03.11 | c | a | a | a | c | Moderate |
| Won et al [124], 2021 | South Korea | 2020.01.20-2020.02.10 | b | c | e | b | a | Moderate |
| Wong et al [125], 2020 | Brunei | 2020.03.09-2020.04.05 | a | a | a | a | a | Strong |
| Wu et al [128], 2021 | China | - | a | c | a | b | c | Moderate |
| Wu et al [127], 2020 | China | 2020.01-2020.02 | c | a | e | a | c | Moderate |
| Wu et al [126], 2020 | China | by 2020.02.18 | a | a | b | a | a | Strong |
| Wu et al [129], 2020 | China | 2020.01.23-2020.02.09 | a | a | e | a | c | Moderate |
| Xiao et al [131], 2020 | China | 2020.01.19-2020.03.03 | c | a | c | a | c | Moderate |
| Xiao et al [133], 2021 | China | by 2020.02.21 | a | c | d | b | a | Moderate |
| Xiao et al [132], 2020 | China | by 2020.02.12 | a | c | c | b | a | Moderate |
| Xiao et al [130], 2020 | China | 2020.01.24-2020.02.16 | d | c | e | b | c | Weak |
| Xie et al [134], 2020 | China | 2020.01.01-2020.02.15 | c | c | e | b | c | Weak |
| Xin et al [135], 2020 | China | 2020.07.10-2021.04.02 | a | c | c | b | a | Moderate |

© 2022 Wu Y et al. *JAMA Network Open.*

| | | | | | | | | |
|---|---|---|---|---|---|---|---|---|
| Xu et al [136], 2020 | China | 2020.01.23-2020.02.18 | a | a | e | a | c | Moderate |
| Xu et al [137], 2020 | China | 2020.01.10-2020.01.26 | c | a | a | a | c | Moderate |
| Yang et al [138], 2020 | China | 2020.01.01-2020.02.20 | a | c | a | b | a | Moderate |
| Yang et al [139], 2021 | China | 2020.01.21-2020.02.25 | c | a | e | a | a | Moderate |
| Yang et al [140], 2020 | China | 2020.01.25-2020.02.08 | a | a | a | a | a | Strong |
| You et al [141], 2020 | China | by 2020.03.31 | b | a | b | a | a | Strong |
| Yu et al [143], 2022 | China | 2020.01.01-2020.12.31 | a | c | e | b | c | Weak |
| Yu et al [142], 2020 | China | by 2020.02.19 | a | a | c | a | a | Strong |
| Zhang et al [146], 2021 | China | 2020.01.27-2020.02.13 | a | a | c | a | a | Strong |
| Zhang et al [144], 2020 | China | 2020.01.19-2020.02.17 | a | c | c | b | a | Moderate |
| Zhang et al [145], 2020 | China | 2020.01.26-2020.02.20 | a | a | b | a | c | Moderate |
| Zhang et al [147], 2021 | China | by 2020.03.11 | a | c | d | a | a | Moderate |
| Zhang et al [148], 2021 | China | - | a | a | a | a | a | Strong |
| Zhao et al [151], 2021 | China | by 2020.04.08 | a | c | a | b | a | Moderate |
| Zhao et al [152], 2020 | China | 2020.01.16-2020.02.17 | c | a | a | a | a | Strong |
| Zhao et al [153], 2021 | China | 2020.01.07-2020.05.17 | a | a | c | a | a | Strong |
| Zhao et al [150], 2021 | China | 2021.05.21-2021.06.18 | a | c | a | b | a | Moderate |
| Zhao et al [149], 2020 | China | 2020.01.21-2020.02.22 | c | c | e | b | c | Weak |
| Zhong et al [154], 2020 | China | 2020.01.21-2020.02.10 | c | a | c | a | a | Moderate |
| Zhu et al [156], 2021 | China | 2020.02.26-2020.04.04 | a | c | c | b | a | Moderate |
| Zhu et al [155], 2020 | China | by 2020.03.26 | b | a | e | a | c | Moderate |
| Zhu et al [157], 2021 | China | 2021.01.02-2021.02.14 | a | c | c | b | a | Moderate |

© 2022 Wu Y et al. *JAMA Network Open.*

**eTable 4.** Characteristics of the Studies Included in the Systematic Review and Meta-analysis

| Author | Time period | Type of design | Method of assessing strain types | Virus strain | Sample size |
|---|---|---|---|---|---|
| Areekal et al [16], 2021 | 2020.06-2020.07 | Cross-sectional study | PCR/Rapid Antigen Test | Wild-type strain | 311 |
| Backer et al [18], 2020 | 2020.01.20-2020.01.28 | Cross-sectional study | | Wild-type strain | 88 |
| Backer et al [17], 2020 | 2021.12.13-2021.12.26 | Cohort study | PCR | Multiple strains | 258 |
| Bao et al [19], 2020 | 2020.01.15-2020.02.06 | Case series | PCR | Wild-type strain | 57 |
| Brandal et al [20], 2021 | 2021.11.26-2021.12.13 | Case series | PCR/WGS | Omicron variant | 81 |
| Bui et al [21], 2020 | 2020.01.23-2020.04.13 | Model study | | Wild-type strain | 19 |
| Chen et al [22], 2020 | 2020.01.24-2020.02.13 | Case series | | Wild-type strain | 18 |
| Covid-Epidemiology Investigation Team [23], 2021 | 2020.12.23-2021.01.31 | Case series | PCR/Sequence alignment and phylogenetic analyses | B.1.160.4 variant | 31 |
| Dai et al [24], 2020 | 2020.01.20-2020.02.29 | Model study | | Wild-type strain | 180 |
| Del Águila-Mejía et al [26], 2022 | 2021.11-2021.12 | Cohort study | PCR/WGS | Multiple strains | 333 |
| Deng et al [27], 2021 | by 2020.03.09 | Cohort study | PCR | Wild-type strain | 43 |
| Deng et al [28], 2020 | by 2020.03.31 | Model study | | Wild-type strain | 198 |
| Denis et al [29], 2021 | 2020.12.15-2020.12.24 | Cross-sectional study | PCR/Rapid Antigen Test | Wild-type strain | 1676 |
| Ding et al [30], 2020 | 2020.01.21-2020.03.10 | Cross-sectional study | PCR | Wild-type strain | 27 |
| Dong et al [31], 2020 | 2020.01.13-2020.02.21 | Cross-sectional study | PCR | Wild-type strain | 16 |
| Du et al [32], 2020 | 2020.01.08-2020.02.05 | Model study | | Wild-type strain | 109 |

© 2022 Wu Y et al. *JAMA Network Open.*

| | | | | | |
|---|---|---|---|---|---|
| Gao et al [34], 2020 | 2020.01.21-2020.02.11 | Cross-sectional study | | Wild-type strain | 24 |
| Gao et al [33], 2020 | 2020.01.20-2020.03.17 | Cohort study | PCR | Wild-type strain | 12 |
| Grant et al [35], 2022 | 2021.05.23-2021.08.13 | Case control study | PCR | Multiple strains | 7553 |
| Guo et al [36], 2020 | 2020.01.15-2020.03.15 | Cross-sectional study | PCR | Wild-type strain | 85 |
| Han et al [37], 2020 | 2020.03.28-2020.04.08 | Case series | PCR | Wild-type strain | 8 |
| Han et al [39], 2020 | 2020.01.20-2020.03.10 | Cross-sectional study 研究 | PCR | Wild-type strain | 226 |
| Han et al [38], 2020 | 2020.01.31-2020.02.16 | Cohort study | PCR | Wild-type strain | 25 |
| Hong et al [40], 2020 | 2020.01.14-2020.03.09 | Model study | | Wild-type strain | 58 |
| Hua et al [41], 2020 | 2020.01.01-2020.02.29 | Cohort study | PCR | Wild-type strain | 43 |
| Huang et al [43], 2020 | 2020.01.08-2020.02.16 | Cross-sectional study 研究 | | Wild-type strain | 734 |
| Huang et al [42], 2020 | 2020.01.22-2020.06.18 | Cohort study | PCR | Wild-type strain | 429 |
| Huang et al [44], 2021 | 2020.01.01-2020.04.08 | Model study | | Wild-type strain | 787 |
| Je et al [45], 2021 | 2020.02-2020.04 | Cohort study | PCR | Wild-type strain | 29 |
| Jeong et al [46], 2020 | 2020.02.22-2020.02.26 | Case series | PCR | Wild-type strain | 25 |
| Jiang et al [47], 2020 | 2020.01.12-2020.02.20 | Case series | PCR | Wild-type strain | 43 |
| Jiang et al [48], 2021 | 2020.01.19-2020.01.23 | Model study | | Wild-type strain | 147 |
| Jin et al [49], 2020 | 2020.01.17-2020.02.08 | Cohort study | PCR | Wild-type strain | 195 |
| Khonyongwa et al [50], 2020 | 2020.03.01-2020.04.18 | Cross-sectional study | PCR | Wild-type strain | 44 |
| Ki et al [51], 2020 | 2020.03.01-2020.06.30 | Model study | | Wild-type strain | 79 |

© 2022 Wu Y et al. *JAMA Network Open.*

| | | | | | |
|---|---|---|---|---|---|
| Kim et al [52], 2020 | 2020.02.04-2020.04.07 | Case series | PCR | Wild-type strain | 7 |
| Kong et al [53], 2020 | 2020.01.08-2020.01.27 | Case series | | Wild-type strain | 10 |
| Kong et al [55], 2020 | 2020.01.22-2020.02.15 | Cohort study | | Wild-type strain | 136 |
| Kong et al [54], 2020 | 2020.01.25-2020.02.20 | Cohort study | PCR/WGS | Wild-type strain | 411 |
| Lai et al [56], 2020 | 2020.01.11-2020.02.10 | Cohort study | PCR/WGS | Wild-type strain | 330 |
| Lau et al [57], 2021 | 2020.01.01-2020.01.29 | Model study | | Wild-type strain | 160 |
| Laval et al [25], 2021 | 2020.02.25-2020.03.04 | Case series | PCR | Wild-type strain | 23 |
| Le et al [58], 2020 | 2020.01-2020.02 | Case series | PCR/WGS | Wild-type strain | 11 |
| Lee et al [59], 2021 | 2021.11.24-2021.12.10 | Case series | PCR | Omicron variant | 80 |
| Lei et al [60], 2020 | 2020.01.22.2020.02.01 | Case series | | Wild-type strain | 7 |
| Leung et al [61], 2020 | 2020.01.20-2020.02.12 | Model study | | Wild-type strain | 90 |
| Li et al [63], 2020 | 2020.01.21-2020.02.09 | Cohort study | PCR | Wild-type strain | 74 |
| Li et al [64], 2022 | 2021.05.21-2021.06.24 | Cross-sectional study | PCR | Delta variant | 103 |
| Li et al [62], 2020 | 2019.12-2020.01 | Cross-sectional study | PCR | Wild-type strain | 10 |
| Linton et al [65], 2020 | by 2020.01.31 | Model study | | Wild-type strain | 158 |
| Liu et al [66], 2021 | 2020.01.20-2020.02.23 | Case series | PCR | Wild-type strain | 6 |
| Liu et al [68], 2020 | 2020.01.20-2020.04.15 | Cohort study | | Wild-type strain | 49 |
| Liu et al [69], 2020 | 2019.12.25-2020.02.05 | Cross-sectional study | | Wild-type strain | 33 |
| Liu et al [70], 2020 | 2020.01.28-2020.04.12 | Cross-sectional study | | Wild-type strain | 27 |
| Liu et al [71], 2020 | 2020.01.22-2020.02.21 | Cohort study | | Wild-type strain | 41 |

© 2022 Wu Y et al. *JAMA Network Open.*

| | | | | | |
|---|---|---|---|---|---|
| Liu et al [72], 2020 | 2020.01.23-2020.02.10 | Cross-sectional study | | Wild-type strain | 44 |
| Liu et al [74], 2021 | 2020.01.01-2020.03.30 | Cross-sectional study | PCR/NGS | Wild-type strain | 57 |
| Liu et al [67], 2020 | by 2020.02.11 | Cross-sectional study | PCR/WGS | Wild-type strain | 431 |
| Liu et al [73], 2020 | 2020.01.20-2020.03.17 | Cohort study | PCR | Wild-type strain | 90 |
| Llaque-Quiroz et al [75], 2020 | 2020.03-2020.05 | Cross-sectional study | PCR/Rapid Antigen Test | Wild-type strain | 33 |
| Mao et al [76], 2020 | by 2020.03.17 | Cross-sectional study | PCR | Wild-type strain | 28 |
| Moazzami et al [77], 2021 | 2020.04 | Case series | | Wild-type strain | 23 |
| Ng et al [78], 2021 | by 2020.03.21 | Case series | | Wild-type strain | 158 |
| Nie et al [79], 2020 | 2020.01.19-2020.02.08 | Cross-sectional study | | Wild-type strain | 2907 |
| Ogata et al [80], 2022 | 2020.07.01-2021.09.16 | Cohort study | PCR/WGS | Multiple strains | 121 |
| Pak et al [81], 2020 | 2019.12.01-2020.03.30 | Model study | | Wild-type strain | 207 |
| Pan et al [82], 2020 | 2020.01-2020.02.20 | Cross-sectional study | PCR | Wild-type strain | 18 |
| Patrikar et al [83], 2020 | by 2020.05 | Model study | | Wild-type strain | 268 |
| Paul et al [84], 2021 | by 2020.11.07 | Model study | | Unknown strain | 211735 |
| Ping et al [85], 2021 | 2020.01.03-2020.03.05 | Cross-sectional study | PCR/WGS | Wild-type strain | 90 |
| Pongpirul et al [86], 2020 | 2020.01.08-2020.04.16 | Cross-sectional study | PCR | Wild-type strain | 83 |
| Pung et al [87], 2020 | 2020.02.03-2020.02.15 | Cross-sectional study | PCR | Wild-type strain | 19 |

© 2022 Wu Y et al. *JAMA Network Open.*

| | | | | | |
|---|---|---|---|---|---|
| Qi et al [88], 2020 | 2020.01.13-2020.03.23 | Case series | PCR | Wild-type strain | 56 |
| Qian et al [89], 2020 | 2020.01.20-2020.02.11 | Case series | PCR | Wild-type strain | 91 |
| Qiu et al [90], 2020 | 2020.01.22-2020.02.12 | Cross-sectional study | | Wild-type strain | 104 |
| Ratovoson et al [91], 2021 | 2020.03.09-2020.07.30 | Case series | PCR | Wild-type strain | 32 |
| Ren et al [92], 2020 | by 2020.01.23 | Cross-sectional study | | Wild-type strain | 98 |
| Samrah et al [93], 2021 | 2020.03.15-2020.06.09 | Cohort study | PCR | Unknown strain | 157 |
| Sanche et al [94], 2020 | 2020.01.15-2020.01.30 | Model study | | Wild-type strain | 24 |
| Shen et al [95], 2020 | 2020.01.08-2020.02.19 | Cross-sectional study | | Wild-type strain | 6 |
| Shen et al [96], 2020 | 2020.01-2020.02 | Case series | | Wild-type strain | 7 |
| Shi et al [97], 2020 | 2020.01.17-2020.03.05 | Cross-sectional study | | Wild-type strain | 38 |
| Shi et al [98], 2020 | 2020.01.18-2020.03.02 | Cross-sectional study | PCR | Wild-type strain | 46 |
| Shiel et al [99], 2021 | 2020.01-2020.06 | Cross-sectional study | PCR | Unknown strain | 100 |
| Shu et al [100], 2020 | by 2020.02.28 | Cross-sectional study | PCR/WGS | Wild-type strain | 32 |
| Song et al [101], 2020 | by 2020.01.26 | Model study | | Wild-type strain | 90 |
| Song et al [102], 2020 | 2020.01.16-2020.01.29 | Cross-sectional study | PCR | Wild-type strain | 22 |
| Song et al [103], 2020 | 2020.02-2020.03 | Cross-sectional study | PCR | Wild-type strain | 17 |
| Su et al [104], 2021 | 2020.01.21-2020.04.18 | Cross-sectional study | PCR | Wild-type strain | 101 |

© 2022 Wu Y et al. *JAMA Network Open.*

| | | | | | |
|---|---|---|---|---|---|
| Sugano et al [105], 2020 | 2020.01.15-2020.03.19 | Case series | PCR | Wild-type strain | 40 |
| Sun et al [106], 2021 | 2020.12.23-2021.01.10 | Case series | PCR | Unknown strain | 19 |
| Sun et al [107], 2020 | 2020.01.24-2020.02.16 | Case series | PCR | Wild-type strain | |
| Sun et al [108], 2020 | 2020.01.20-2020.02.15 | Case series | PCR | Wild-type strain | 55 |
| Sun et al [109], 2021 | 2020.06-2020.07.10 | Case series | PCR/WGS | B.1.1 variant | |
| Sun et al [110], 2020 | 2020.02 | Case series | PCR | Wild-type strain | |
| Tan et al [111], 2020 | 2020.01.23-2020.04.02 | Cross-sectional study | PCR | Wild-type strain | 164 |
| Tanaka et al [112], 2022 | 2022.01.01-2022.01.28 | Cross-sectional study | PCR/Time perid | Multiple strains | 77 |
| The SARS-CoV-2 variant with line [113], 2021 | 2020.12-2021.01 | Case series | PCR/WGS | Beta variant | 10 |
| Tian et al [114], 2020 | 2020.01.20-2020.02.10 | Cohort study | PCR | Wild-type strain | 262 |
| Tindale et al [115], 2020 | 2020.01.21-2020.02.22 | Cross-sectional study | | Wild-type strain | 135 |
| Viego et al [116], 2020 | 2020.03.20-2020.05.08 | Cross-sectional study | | Unknown strain | 15 |
| Wang et al [158], 2021 | by2020.02.26 | Case series | PCR | Wild-type strain | 14 |
| Wang et al [117], 2020 | 2020.01.23-2020.02.20 | Case series | PCR | Wild-type strain | 62 |
| Wang et al [118], 2020 | 2020.01.01-2020.04.03 | Cross-sectional study | PCR/NGS | Wild-type strain | 219 |
| Wang et al [119], 2020 | 2020.01.05-2020.02.12 | Case series | PCR/WGS | Wild-type strain | 14 |
| Wang et al [120], 2020 | 2020.01.20-2020.02.10 | Case series | PCR/WGS | Wild-type strain | 275 |
| Wei et al [123], 2021 | 2020.01.19-2020.03.01 | Case series | | Wild-type strain | 28 |
| Wei et al [122], 2020 | 2020.01.27-2020.03.11 | Case series | PCR/NGS | Wild-type strain | 71 |

© 2022 Wu Y et al. *JAMA Network Open.*

| | | | | | |
|---|---|---|---|---|---|
| Won et al [124], 2021 | 2020.01.20-2020.02.10 | Model study | | Wild-type strain | 28 |
| Wong et al [125], 2020 | 2020.03.09-2020.04.05 | Cross-sectional study | PCR | Wild-type strain | 15 |
| Wu et al [128], 2021 | - | Cross-sectional study | PCR/WGS | Unknown strain | 39 |
| Wu et al [127], 2020 | 2020.01-2020.02 | Case series | PCR | Wild-type strain | 14 |
| Wu et al [126], 2020 | by 2020.02.18 | Case series | PCR | Wild-type strain | 19 |
| Wu et al [129], 2020 | 2020.01.23-2020.02.09 | Case series | PCR | Wild-type strain | 41 |
| Xiao et al [131], 2020 | 2020.01.19-2020.03.03 | Case series | PCR | Wild-type strain | 44 |
| Xiao et al [133], 2021 | by 2020.02.21 | Cross-sectional study | PCR | Wild-type strain | 217 |
| Xiao et al [132], 2020 | by 2020.02.12 | Cross-sectional study | | Wild-type strain | 2555 |
| Xiao et al [130], 2020 | 2020.01.24-2020.02.16 | Cross-sectional study | PCR | Wild-type strain | 279 |
| Xie et al [134], 2020 | 2020.01.01-2020.02.15 | Case series | PCR | Wild-type strain | 21 |
| Xin et al [135], 2020 | 2020.07.10-2021.04.02 | Model study | PCR | Unknown strain | 177 |
| Xu et al [136], 2020 | 2020.01.23-2020.02.18 | Case series | PCR | Wild-type strain | 19 |
| Xu et al [137], 2020 | 2020.01.10-2020.01.26 | Case series | | Wild-type strain | 56 |
| Yang et al [138], 2020 | 2020.01.01-2020.02.20 | Case series | | Wild-type strain | 325 |
| Yang et al [139], 2021 | 2020.01.21-2020.02.25 | Case series | | Wild-type strain | 74 |
| Yang et al [140], 2020 | 2020.01.25-2020.02.08 | Case series | PCR | Wild-type strain | 10 |
| You et al [141], 2020 | by 2020.03.31 | Model study | | Wild-type strain | 169 |
| Yu et al [143], 2022 | 2020.01.01-2020.12.31 | Model study | | Unknown strain | 211 |

© 2022 Wu Y et al. *JAMA Network Open.*

| | | | | | |
|---|---|---|---|---|---|
| Yu et al [142], 2020 | by 2020.02.19 | Cross-sectional study | | Wild-type strain | 57 |
| Zhang et al [146], 2021 | 2020.01.27-2020.02.13 | Case series | | Wild-type strain | 10 |
| Zhang et al [144], 2020 | 2020.01.19-2020.02.17 | Cross-sectional study | | Wild-type strain | 49 |
| Zhang et al [145], 2020 | 2020.01.26-2020.02.20 | Cohort study | PCR | Wild-type strain | 6 |
| Zhang et al [147], 2021 | by 2020.03.11 | Cross-sectional study | | Wild-type strain | 139 |
| Zhang et al [148], 2021 | - | Cross-sectional study | | Unknown strain | 353 |
| Zhao et al [151], 2021 | by 2020.04.08 | Model study | | Wild-type strain | 254 |
| Zhao et al [152], 2020 | 2020.01.16-2020.02.17 | Case series | | Wild-type strain | 45 |
| Zhao et al [153], 2021 | 2020.01.07-2020.05.17 | Model study | | Unknown strain | 102 |
| Zhao et al [150], 2021 | 2021.05.21-2021.06.18 | Cross-sectional study | PCR/WGS | Delta variant | 39 |
| Zhao et al [149], 2020 | 2020.01.21-2020.02.22 | Case series | | Wild-type strain | 26 |
| Zhong et al [154], 2020 | 2020.01.21-2020.02.10 | Case series | | Wild-type strain | 62 |
| Zhu et al [156], 2021 | 2020.02.26-2020.04.04 | Cross-sectional study | | Wild-type strain | 197 |
| Zhu et al [155], 2020 | by 2020.03.26 | Cross-sectional study | | Wild-type strain | 245 |
| Zhu et al [157], 2021 | 2021.01.02-2021.02.14 | Model study | | Unknown strain | 869 |

© 2022 Wu Y et al. *JAMA Network Open.*

eFigure 1. Funnel Plot with a 95% Confidence Interval for Included Studies in the Meta-analysis

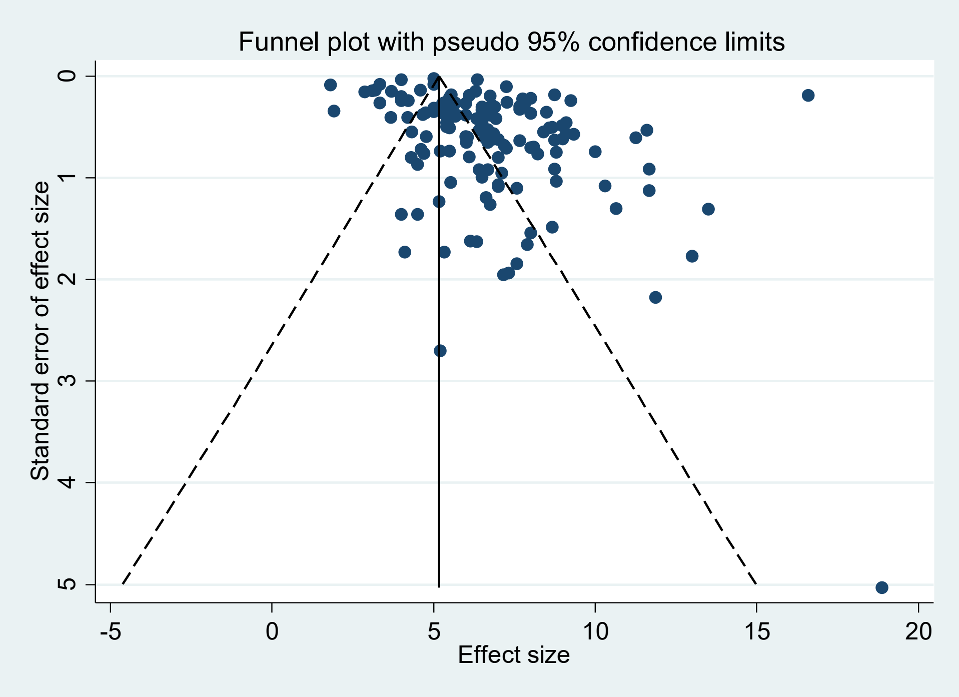


© 2022 Wu Y et al. *JAMA Network Open.*

eFigure 2. Forest Plot for the Meta-analysis of Incubation Period for COVID-19 Caused by Wild-type Strain

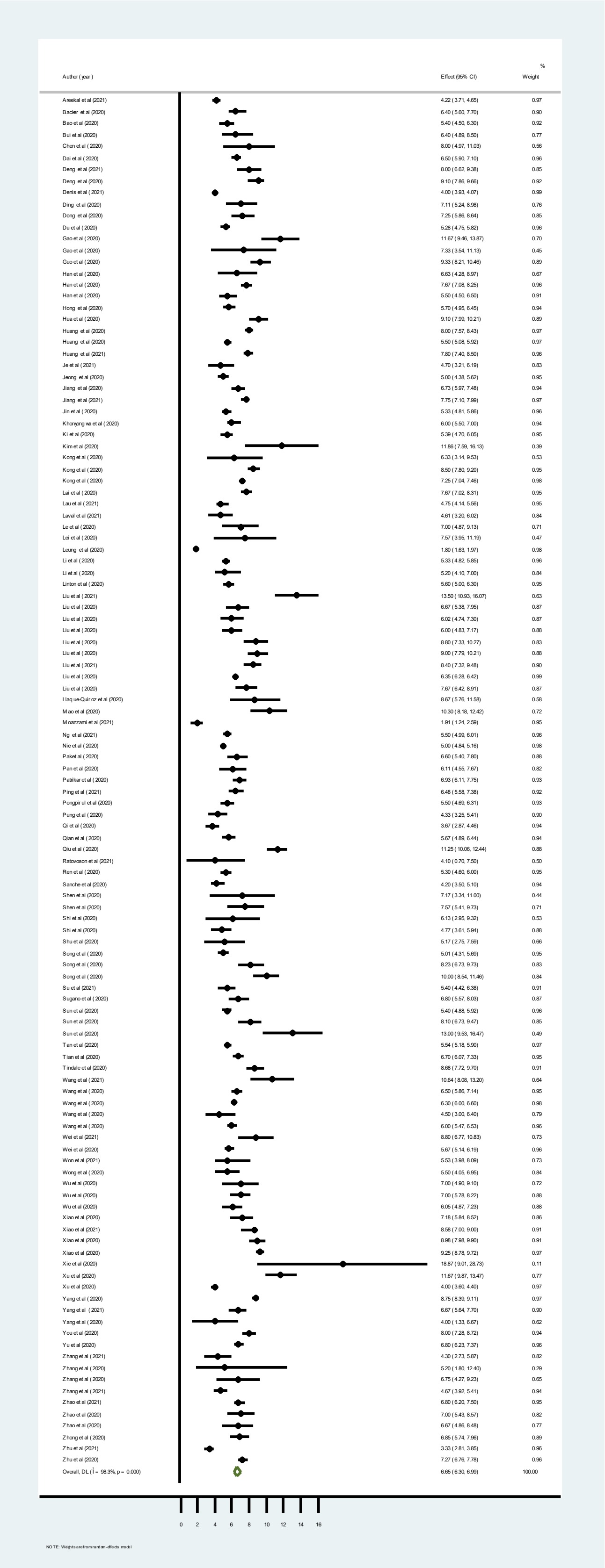


© 2022 Wu Y et al. *JAMA Network Open.*
